# Collaboration for rare disease drug discovery research

**DOI:** 10.12688/f1000research.5564.1

**Published:** 2014-10-31

**Authors:** Nadia K. Litterman, Michele Rhee, David C. Swinney, Sean Ekins

**Affiliations:** 1 Collaborative Drug Discovery, Inc., Burlingame, CA, 94010, USA; 2 National Brain Tumor Society, Newton, MA, 02458, USA; 3 Institute for Rare and Neglected Diseases Drug Discovery (iRND3), Mountain View, CA, 94043, USA; 4 Collaborations in Chemistry, Fuquay Varina, NC, 27526, USA; 5 Phoenix Nest Inc., Brooklyn, NY, 11215, USA; 6 Hereditary Neuropathy Foundation, New York, NY, 10016, USA; 7 Hannah's Hope Fund, Rexford, NY, NY 12148, USA

**Keywords:** rare disease, patient advocacy, drug discovery, Twitter

## Abstract

Rare disease research has reached a tipping point, with the confluence of scientific and technologic developments that if appropriately harnessed, could lead to key breakthroughs and treatments for this set of devastating disorders. Industry-wide trends have revealed that the traditional drug discovery research and development (R&D) model is no longer viable, and drug companies are evolving their approach. Rather than only pursue blockbuster therapeutics for heterogeneous, common diseases, drug companies have increasingly begun to shift their focus to rare diseases. In academia, advances in genetics analyses and disease mechanisms have allowed scientific understanding to mature, but the lack of funding and translational capability severely limits the rare disease research that leads to clinical trials. Simultaneously, there is a movement towards increased research collaboration, more data sharing, and heightened engagement and active involvement by patients, advocates, and foundations. The growth in networks and social networking tools presents an opportunity to help reach other patients but also find researchers and build collaborations. The growth of collaborative software that can enable researchers to share their data could also enable rare disease patients and foundations to manage their portfolio of funded projects for developing new therapeutics and suggest drug repurposing opportunities. Still there are many thousands of diseases without treatments and with only fragmented research efforts. We will describe some recent progress in several rare diseases used as examples and propose how collaborations could be facilitated. We propose that the development of a center of excellence that integrates and shares informatics resources for rare diseases sponsored by all of the stakeholders would help foster these initiatives.

## Introduction

Although each rare disease affects less than 200,000 individuals in the United States, in aggregate, rare diseases affect 6–7% of the population ^1^. As less than 10% of these patients can be presently treated, this remains a very large unmet medical need ^2^. According to the National Organization for Rare Disorders (NORD), there are only 250 treatments for the nearly 7,000 rare disorders, impacting nearly 30 million Americans. Eighty percent of these diseases have a genetic origin ^1, 3^. Most rare diseases are caused by mutations in a single gene, such as an enzyme deficiency (like α-galactosidase A in Fabry’s Disease). Other diseases, such as Charcot-Marie-Tooth (CMT), have multiple genetic causes ^4^. In either case, the knowledge of the genetic basis of the disease can be quite illuminating and lead to therapeutic development efforts ^3^.

In metabolic disorders such as the lysosomal storage diseases, the development of biologics such as enzyme replacement therapy has been quite fruitful. In rare cancers, the knowledge of mutated kinases has led to the development of specific, potent small molecule inhibitors ^5, 6^. There are many diseases with approved drugs developed by a knowledge of genetics such as: myelofibrosis, non-small cell lung cancer (NSCLC), late stage melanoma, chronic myelogenous leukemia (CML), Gaucher's disease, Pompe's disease, hyperphenylalaninemia, Hunter syndrome, mucopolysaccharidosis (MPS) VI, MPS I, Fabry's disease, Type I tyrosinemia, hyperammonemia due to N-acetylglutamate synthase (NAGS) deficiency, cystic fibrosis, hereditary angioedema (HAE), cryopryin-associated periodic syndromes, and paroxysmal nocturnal hemoglobinuria ^3^.

Developing novel therapeutics is always a risky and difficult endeavor ^7, 8^, and the process of drug discovery for rare diseases is marked by unique challenges. Rare diseases represent an example of the power of individualized therapies, but under the current paradigm the trade-off is that these are incredibly expensive ^9, 10^. So there is an urgent need to discover ways to develop therapies more cost-effectively ^11^. Using our perspectives as rare disease researchers and patient advocates with experience of facilitating collaborations, we will use the diseases we have most knowledge of to outline the challenges that we see and offer some proposed solutions in this opinion article.

## Getting connected

Rare diseases, by definition, have very small numbers of patients (sometimes in the low tens to hundreds) that are often dispersed and disconnected globally. This is problematic for understanding the natural history of the disease, identifying the underlying mechanisms, and recruiting patients for clinical trials ^3^. Before a therapy can be developed, the natural history needs to be understood so that the clinical trials can attempt to show a positive outcome with the disease in question. Despite the inherent difficulties, recent trends and technological developments, including in genomics, collaboration, and even social media, can be harnessed to the advantage of rare disease patients and researchers.

The connectivity and network-building enabled by the internet is especially important for rare disease patients and caregivers during diagnosis and treatment. Even well-informed individual physicians are unlikely to have experience with all given rare conditions, making diagnoses challenging. Despite the importance for support and knowledge sharing, it is extremely unlikely for rare disease patients to find one another through traditional methods like face to face networking, conferences, newspaper and magazine articles, etc., especially since the Health Insurance Portability and Accountability Act (HIPAA) privacy rules make it difficult ^12, 13^ to share information. Recent technological advancements have helped to reduce the barriers for doctors, caregivers, and patients to reach out to find one another. Social network sites such as Sermo ^14^ and Doximity ^15^ enable physicians to crowdsource a diagnosis. In addition, patients can find one another through websites and social media. For example, many rare disease groups set up public or private Facebook pages. Some are totally open and write regular blog posts to communicate their activities and goals, or what they have done to increase awareness, fund-raise, or look for a cure. In other cases they may be private sites for caregivers to use them to share their experiences. Both approaches enable families with very rare diseases to connect and then build momentum from there ^16, 17^. Many rare disease advocates are also users of Twitter as a tool to highlight articles of interest, promote their fundraising events, or just share their experiences ( Table 1). Overall, this increased connectivity benefits patients, trying to identify the source of their symptoms and understand their recent diagnosis. In addition, such connected patient networks can also lead to key research breakthroughs, such as defining the genetic origin of the disease, understanding the natural history, defining biomarkers, and recruiting patients for clinical registries, natural history studies and clinical trials. A useful side effect of this social networking is to raise the overall level of awareness amongst the population that was previously not familiar with rare diseases.

**Table 1.** List of rare disease related Twitter users. (Also see http://www.totalbiopharma.com/2013/07/01/top-50-social-media-influencers-orphan-drugs-rare-disease/ and http://moderators.rareconnect.org/social-media-case-studies/raredisease-patient-advocates-follow-these-25-twitter-accounts/).

@RareDR	@alsadvocacy
@RareDiseases	@CheckOrphan
@SDFatPhRMA	@eurordis
@savingcase	@AfternoonNapper
@JJB4CURE	@HurtBlogger
@RareVoices	@bensfriends
@RareDayUS	@alanROYGBIV
@FMDChat	@_chrisco
@ncats_nih_gov	@CureTheProcess
@ORDR	@michelerhee
@GlobalGenes	@lisamjarvis

## The role of patient advocacy organizations

Historically, industrial sponsors of research and clinical trials see the rare disease space as riskier, and less profitable than more common diseases. The perception around profit has shifted as rare disease blockbuster drugs (such as Vertex’s Kalydeco™ for cystic fibrosis) make headlines. Still, key decision-makers within biopharmaceutical companies continue to be hesitant about pursuing rare disease indications due to the perceived risk. When the average drug costs $500 million–$1 billion (or more) and takes 15–20 years to develop ^7, 8^, companies want to reduce the likelihood of failure and increase the potential revenue as much as possible. Hence the rationale behind rare disease drugs that cost upwards of $100,000 per year. To be simplistic, the calculation considers whether the amount of money spent to get a drug to market will be less than the amount of money received in revenue over the lifetime of the patent exclusivity of the drug. The equation tends to come out on the wrong side for rare diseases, in large part because many of the attempts to get orphan drugs to market have failed, as evidenced by the lack of launched drugs. These failures increase the perception of riskiness of the space, which means that, at a minimum, companies are less likely to invest in programs focused on a rare disease.

One approach to solving this catch-22 problem is for patient advocacy groups to collaborate with academia, government, not-for profits and biopharmaceutical companies to increase incentives for investment in rare-disease-specific programs. Patient advocacy organizations play a role ^9, 18, 19^ here because they can be uniquely strategic and creative to reduce risk, bring patients together for researchers and drive research forward.

### Reducing financial risk of investment

At a base level, risk can be reduced if the cost of entering the disease area can be lowered, reducing the initial financial risk. When a company is deciding whether to pursue a specific indication for a disease, it is competing against others that may have existing pre-clinical models and clinical trial networks. The investment required is therefore often higher in rare diseases because the infrastructure does not exist. The barrier to entry can be mitigated through the support of and collaboration with disease-specific patient advocacy groups. Large organizations such as the Michael J. Fox Foundation for Parkinson’s Disease Research and the Cystic Fibrosis Foundation have used a modified venture philanthropy model. This allows them to invest in and provide research grants to biotechnology companies who have designated programs in their specific disease areas. These investments and grants become true collaborations over time as these large patient advocacy groups provide disease expertise, access to key opinion leaders and patients, and clinical trial recruitment support. The key word here is time. These collaborations can last over a decade. Obviously, however, not all rare disease groups have the financial resources to invest the funding required to significantly reduce risk which at this level is likely in the $10–100 millions range. Also, many groups are trying to discover therapies in a shorter time if possible. However, patient groups do not have to invest at this level to have an impact. Smaller investments in the low tens of thousands of dollars can have an impact in funding science ^9^, providing the seed funding to develop an assay or animal model or even make a compound for testing. These efforts may more frequently be targeted to academia as long as the overhead costs can be kept to a minimum or avoided.

Brain cancer offers an example of where a company was able to spread the financial risk across a number of patient advocacy groups. Tocagen applied for and received research grants from three major brain tumor patient advocacy groups: National Brain Tumor Society, Accelerate Brain Cancer Cure and American Brain Tumor Association. Although the financial support was helpful, the company also benefited by having the support of these patient advocacy organizations as it recruited for clinical trials. Typically, because of the small patient population numbers, clinical trial recruitment is a huge issue in rare disease clinical trials, but Tocagen has not suffered the traditional patient recruitment problems.

### Reducing regulatory risk

Other collaborative approaches that do not require as much financial outlay can also be successful at encouraging companies to build or to further develop specific programs. These are typically more policy- and advocacy-based and are often areas in which only patient advocacy groups or other not-for-profits can lead. Existing policies that increase incentives to discover new medical entities for rare diseases include the Orphan Drug Act, pediatric priority review vouchers, and extended patent exclusivity upon the inclusion of a pediatric indication.

Patient advocacy groups ^18^ can also use their passion and experience to clarify some of the ambiguity in the regulatory environment for rare disease drug evaluations. Because there have been so few successes in the orphan drug space, drug companies have limited precedents to follow as they design their clinical trials and navigate the Food and Drug Administration (FDA). Typically, the FDA speaks to trial sponsors only within the context of a specific application, which is less of an issue when there have been recent or multiple approvals in a disease because the trial design and endpoints are clear. Companies will err on the conservative side and design trials with larger numbers of patients using endpoints that often take longer (for example, using overall survival instead of a surrogate endpoint).

One approach to clarifying this regulatory uncertainty is for the patient advocacy community to collaborate with key stakeholders to have open discussions about trial design and endpoints. For example, the Jumpstarting Brain Tumor Drug Discovery Coalition (comprised of the National Brain Tumor Society, the Society for Neuro-oncology, the Musella Foundation, and Accelerate Brain Cancer Cure) has hosted two workshops to discuss alternative and surrogate endpoints for clinical trials. FDA staff, trial sponsors, clinicians and scientists, clinical trial designers, and patients have all been actively involved in working with the patient advocacy coalition to identify endpoints and trial designs that will reduce the time and money required to run a clinical trial in brain cancer. Although the work is still ongoing, the FDA has pointed to this collaboration as an example for other patient advocacy groups to follow, and trial sponsors have enthusiastically participated. Indeed, this approach is being replicated by many rare diseases on an individual basis. Perhaps if all the different rare disease groups collaborated and had these discussions at one time, there could be synergistic effects in terms of prior experience, cost effectiveness and time-savings.

### Increased financial incentive: making the business case

The previous examples of cross-sector collaboration all require either significant financial investment and/or labor outlays. Many rare disease patient advocacy organizations lack substantial funding resources, staff numbers, or even the experienced individuals needed to coordinate collaborations, which limits the options for collaborating with biopharmaceutical companies to encourage greater investment in rare diseases. However, there is an option that is less labor- or finance-intensive: advocating directly to and within the company to support the development of internal programs in rare diseases and providing disease expertise and information.

The National Brain Tumor Society, for example, has collaborated with two biotechnology companies to help them launch brain-cancer-specific programs internally. For both companies, there were a series of meetings and presentations by the patient advocacy group in order to educate the company about the following:

- Unmet need
- Current research landscape
- Treatment paradigm
- Market potential/size

One of the companies is now collaborating with two of the National Brain Tumor Society’s funded researchers, and the other is funding a pilot program in brain cancer. The companies are leveraging their expertise in specific technology to adapt and optimize it for brain cancer treatment. These are pre-clinical programs, and it is anticipated that the relationships and collaborations between the National Brain Tumor Society and the biotechnology companies will continue throughout the development process. It is exciting to see novel technologies applied to a new area where the companies are taking the additional step of tailoring them to the unique needs of the biology of the disease and the patient population. Each company required a different approach, and all collaborations have to be structured to be sensitive to the needs and expectations of each. The passion and commitment of the patient advocacy groups involved were the key drivers in each case. These highly innovative programs have flourished because of the rare disease patient community. It is expected that continued innovation in identifying opportunities will allow further future successes.

### Opportunities in rare disease

Given the current funding environment for academic and startup researchers, it is interesting to note that there may be potential for a surge in rare disease interest and investment. It may seem counterintuitive, but we can look to the 2008 recession for the rationale. In a lean environment where resources are scarce (i.e., the economy is in recession), we often see a boom in startups, as in post-2008 ^20^. High unemployment and limited options led some to be more willing to take on risks such as starting their own business. In short, their opportunity cost has lowered. Similarly, with more academic researchers competing for shrinking federal funding resources, taking on the "risk" of investigating a rare disease indication, which traditionally would have been unappealing due to the lack of resources or a clear career path, has become more desirable. As many trained scientists leave academia, this may be an opportunity to draw their attention and expertise to rare diseases through resources like patient advocacy groups which in turn make some funding available. The decrease in traditional opportunities can then be coupled with the lower cost of getting started in rare disease research. For example, because less research has been done in these orphan areas, some of the most basic (and less expensive) research has yet to be performed. Initial genomic sequencing and analysis for mutations, for example, is cheaper and faster than the more advanced work that is the initial starting point in other more common diseases. Rare disease advocates often must be more imaginative and innovative in order to leverage their limited resources, and in this case, brain power (in the form of brilliant researchers) is a resource that the rare disease community can leverage by presenting a strong case for the career and research opportunities available by focusing in an orphan area.

### Doing more with less—sharing resources

An additional approach for small rare disease foundations is to pool their resources, and that could be at the level of organizational staff for fund raising or at the scientific level. For example, one experienced scientific consultant could oversee the science collaborations for multiple distinct organizations dealing with the same or different rare diseases. This not only has cost savings but also the potential to see synergies across projects and research. This may increase the potential for serendipitous discovery that might synergize the overall research goals for multiple diseases. Obviously, there need to be boundaries to respect the intellectual property of groups involved, but the benefits may outweigh the risks.

## A coordinated research effort

From our own experience of working as researchers or facilitators of collaborations between different groups working on rare diseases, we will now describe some of the results of ongoing efforts. The aim is to give the reader some understanding of the breadth of technologies applied and the number of approaches being worked on simultaneously. Recent examples suggest that through collaborative efforts, more progress can be made.

### Spinal Muscular Atrophy

Spinal Muscular Atrophy (SMA) is a childhood-onset, neurodegenerative disorder that is characterized by the loss of motor neurons and affects approximately one in 11,000 people. The disease has a range of clinical presentations, which are categorized into four types ^21, 22^. Type I, the most severe form that represents approximately 60% of cases, is diagnosed prior to six months of age, and patients do not gain the ability to sit. In 1995, researchers uncovered the genetic basis of the disorder, which accounts for at least 95% of cases, mutations in the Survival of Motor Neuron1 (SMN1) gene ^23^. In addition to SMN1, humans have a variable number of copies of the SMN2 gene, which differs from SMN1 by a single nucleotide, and leads to a change in the splicing pattern, resulting in a truncated form of the protein that is quickly degraded ^24, 25^. The severity of SMA is determined by the number of copies of SMN2, with type I patients having fewer copies. This knowledge allowed for the development of animal models ^26– 28^ and drug discovery assays based on splicing modification and the levels of SMN ^29^. In addition, basic science understanding such as the function of the SMN protein could be probed. Answers to important questions for translational decisions, like where in the body SMN levels must be increased (both in neurons and peripherally) and when treatment is required for response (early intervention is better, but rescue is possible after onset of symptoms) have also been addressed ^30, 31^. Despite the complexity of the SMN protein and the disease pathology, with a clear directive in mind such as to increase levels of SMN by inducing transcription, changing splicing, or preventing protein degradation, the research community has many interesting findings to date which have resulted in five promising drug candidates in clinical trials and at least 11 preclinical programs ^29^. Four out of the five clinical candidates are directly targeting SMN expression, either through gene therapy or modulation of SMN2 transcription or splicing with small molecules or an antisense oligonucleotide (ASO) ^30, 32– 37^ ( Table 2). The furthest along of these candidates is the ISIS-SMN _Rx_ ASO, being developed by ISIS Pharmaceuticals and Biogen-IDEC, which is currently in a Phase III trial ^38^.

SMA research in academia and in industry has been strongly supported and guided by the SMA Foundation ^39^ and CureSMA ^40^ (formerly known as Families of SMA Foundation), which both coordinated research efforts, fostered collaborations, enticed biopharma companies, and developed an extensive patient network for clinical trials. Through these foundations, patient involvement in research was critical for genomic studies, understanding the natural history of SMA, development of induced pluripotent stem cells for disease modeling, clinical trials, and identifying biomarkers. Collaborative partnerships between academia, government, pharmaceutical companies, and non-profits accelerated efforts in compound screening on biochemical and cellular assays, animal testing, and other aspects of drug development, have led to the creation of a robust pipeline over a fifteen year period.

**Table 2.** Drug candidates in clinical trials for the treatment of SMA.

SMA Drug Candidates	Company	Category	Mechanism	Clinical Trials
olesoxime	Trophos	Small molecule	Neuroprotectant	Completed
RG3039	Pfizer	Small molecule	DcpS inhibitor, SMN2 promoter activity	Completed Phase Ia and IB safety
ISIS-SMN _Rx_	Isis Pharmaceuticals and Biogen Idec	Antisense oligonucleotide (ASO)	SMN2 Splicing modulator	Beginning Phase III
RG7800	Roche, PTC Therapeutics, SMA Foundation	Small molecule	SMN2 Splicing modulator	Beginning Phase I
chariSMA	AveXis, Nationwide Children’s Hospital in Columbus, OH	Intraveneously delivered AAV9/SMN1 gene transfer therapy	Gene therapy	Phase I

### Charcot-Marie-Tooth

CMT affects approximately one in 2500 Americans ^41^. Patients usually have muscle weakness, which results in difficulty walking and gripping objects and progresses to foot and hand deformities, decreased reflexes, and bilateral foot drop ^42^. In most cases, the cause is genetic but it can also be induced by other factors such as certain chemotherapy drugs. The Peripheral Myelin Protein 22 (PMP22) gene duplication predominantly causes the most common form of CMT called CMT1A ^4, 43^. There is no treatment for any of the CMTs for which symptoms usually present in the first two decades of life ^44^.

Despite discovery of the causal gene duplication in 1991, the first high throughput screen (HTS) targeting PMP22 was not published until 21 years later in 2012 ^45^. Thus, CMT1A is one of many rare disorders where fundamental discoveries in academia progress slowly towards the therapeutic development. Still, the pipeline for CMT1A looks quite promising with Pharnext announcing a phase III clinical trial for PXT-3003 (a combination therapy of FDA-approved components baclofen, naltrexone and sorbitol) in 2014–2015 ^46, 47^. If the Phase III clinical trial is ultimately successful, this could be the first treatment to market. Still, success is not a given, as success rates for investigational drugs in phase III trials from a recent analysis was 60.1% ^48^. The pharmaceutical company Addex announced a preclinical study of ADX71441, a GABA-B receptor (GABA-BR) positive allosteric modulator (PAM) compound ^49^. Most recently, researchers at the Max Planck Institute (Germany) announced how Neuregulin-1 might represent a promising approach to therapy ^50^. These latter two therapies are likely many years from the clinic.

There are two foundations focused on developing treatments for CMT. The first is the Hereditary Neuropathy Foundation (HNF) ^51^. This group has funded a number of model systems to further research, including the development of a high content screen of over 25,000 compounds for CMT1A (PMP22) and CMT1E (point mutation), the establishment of transgenic CMT1A rat models, a CMT2A (MFN2 mutation) mouse model for testing therapeutics, and a CMT2A zebrafish model for screening. HNF also developed a global registry for inherited neuropathies ^19^. Recently HNF partnered with Pharnext to raise awareness of CMT1A in preparation for their phase III clinical trial. The Charcot-Marie-Tooth Association ^52^ has also raised funds to develop laboratory models and assays at academic partners, perform HTS screening at the National Institutes of Health (NIH) and with pharmaceutical companies (it recently announced a partnerships with Genzyme and Addex). To date the most advanced work has focused on PMP22 for CMT1A. The CMTA has also set up a relationship with a contract research organization to perform drug testing in laboratory models of CMT1A. This foundation also funds work on other CMT forms but this appears to be at an earlier stage than for CMT1A.

The Inherited Neuropathy Consortium Rare Diseases Clinical Research Network (RDCRN) is an NIH collaboration between CMT researchers. Over the past five years, with funding likely in excess of $5 million, this network has focused on determining the natural history of CMT through clinical ( https://www.rarediseasesnetwork.org/INC/studies/index.htm) projects and may be engaged in the future for testing treatments.

At first glance this represents an incredible amount of activity for CMT, but it is worth also considering that there have been considerable failures such as the use of high-dose ascorbic acid for CMT1A ^53, 54^. The heavy focus on PMP22 is a risk given the heterogeneity of the disorder, and this could be mitigated in some way by more collaboration between researchers and foundations to avoid potential for overlap and also explore new approaches. Screening more compounds against PMP22 is likely not going to lead to more insights, and learning from the data already generated via computational modeling would perhaps be beneficial. CMT research is not unique amongst rare diseases in having trouble translating discoveries in the lab into the clinic. It is clear that millions of dollars can be invested by both foundations and the NIH with no guarantee of a treatment resulting from it. Rare disease patients and foundations need to have realistic expectations of the length of time it takes to go from a HTS to the clinic.

### Giant Axonal Neuropathy

Giant Axonal Neuropathy (GAN) is a recessively inherited condition that results in progressive nerve death ^55^, and it has been reviewed previously ^9, 19^. GAN may be closely related to Charcot-Marie-Tooth Type 2 (CMT Type 2) ^56^, and some pathological factors are also hallmarks of amyotrophic lateral sclerosis (ALS or Lou Gehrig’s Disease) ^57^, CMT 2E ^58^, Alzheimer’s disease, Parkinson’s disease, diabetic neuropathy, SMA, as well as other diseases ^59^. A parent/patient led foundation, Hannah’s Hope Fund (HHF) ^60^ has raised over $5 million to fund the development of a gene therapy ^61^. In addition, they are also funding a postdoc at National Center for Advancing Translational Sciences (NCATS) to develop an assay and screen compounds. This illustrates what can be achieved in a short period of time by promoting collaboration between different academic and government research groups ^9, 19^ and perhaps represents a model that other groups could emulate.

### Sanfilippo syndrome

Sanfilippo syndrome (mucopolysaccharidosis type III; MPS III) is a devastating neurodegenerative lysosomal storage disorder of childhood. The cause of MPS III is an inherited mutation in one of four enzymes required to catabolize heparan sulfate (HS). The four subtypes of the disease are defined by the enzyme deficiency: MPS III type A (heparan N-sulfatase); MPS III type B (α-N-acetylglucosaminidase); MPS III type C (heparan sulfate acetyl CoA: α-glucosaminide N-acetyltransferase, HGSNAT); and MPS III type D (N-acetylglucosamine 6-sulfatase) ^62^. All subtypes of MPS III have similar clinical phenotypes with onset in infancy or early childhood: progressive and severe neurological deterioration, hearing loss, and visceral manifestations ^62^. There is currently no cure or effective treatment available for MPS III. There are however many therapies in early development ( Table 3), including gene therapies, enzyme replacement, chaperone and substrate reduction therapeutics. With Sanfilippo MPSIII Type A there is currently a large focus on gene therapy evaluating different vectors (adeno-associated virus (AAV) e.g. AAV5, AAV9, AAVrh.10 etc) across many different groups. Less research appears to be focused on types C, D ( Table 3). Due to the limited pool of funding for this disorder, enhanced collaboration may prevent unnecessary redundancies and broaden the impact of the ongoing research efforts as well as make the investments go further.

**Table 3.** A list of some examples of treatments under development and the research groups involved for Sanfilippo Syndrome. This is by no means exhaustive and we are aware of other efforts, but these are not public knowledge in many cases. (This table is an updated version of that found at https://www.rareconnect.org/en/community/sanfilippo-syndrome/article/current-sanfilippo-research-programs-in-the-clinical-stage).

Sanfilippo Syndrome Type	Treatment	Company Group
A	Gene Therapy SAF-301 completed phase I/II	Lysogene
A	Gene therapy	Nationwide Children’s Hospital/ Abeona Therapeutics
A	Gene therapy	Esteve
A	Enzyme replacement therapy	Shire
A	Stem cells	Univ. Manchester
A	Substrate reduction therapy Genistein	Univ. Manchester
B	Gene therapy ongoing phase I/II	Pasteur Inst.
B	Gene therapy	Nationwide Children’s Hospital/ Abeona Therapeutics ^97^
B	Enzyme replacement therapy	Biomarin/Synageva
B	Substrate reduction therapy Genistein	Univ. Manchester
C	Chaperone	Univ. Montreal ^98^
C	Gene Therapy	Univ. Manchester
	Substrate reduction therapy Genistein	Univ. Manchester
D	Enzyme replacement therapy	LaBioMed/Phoenix Nest ^99^

### A broad impact

The primary goal of rare disease research is to find a cure or treatment strategy for the disorder in question, but rare diseases offer a glimpse into the roles of genes and proteins in human disease pathogenesis in general. The therapeutics developed to treat rare disorders may also be useful in treating additional disorders whether rare or common. This has proven to be true for drugs developed for rare cancer indications. For example, imatinib (Gleevec ^®^) was originally approved for Philadelphia chromosome positive CML, which contains the oncogenic BCR-ABL tyrosine kinase mutation ^63, 64^. Imatinib is a well-absorbed drug with activity against multiple tyrosine kinases beyond BCR-ABL, including c-KIT and PDGFRA ^65, 66^. Due to these other activities, imatinib is effective at treating gastrointestinal stromal tumors (GIST) which are dependent on c-KIT, many other cancers, and steroid refractory Graft-versus-Host disease which requires PDGFRA activity ^67^. Thus drugs developed for one rare disease can serve broader roles based on related biological mechanisms.

## Software for collaborations

There appears to be increased interest in scientific collaborations on a large scale and developing a software to facilitate this ^68^. For rare diseases, collaborations even on a small scale could have real impact. Instead of scientists hoarding their data, we could remove unnecessary duplication and speed development.

### Collaborative Drug Discovery (CDD) vault

Research collaborations are seen as important for drug discovery to speed up biomedical research, reduce costs, and prevent unnecessary repetition of experiments ^69^. There are however considerable intellectual property (IP) concerns to be overcome when sharing data ^70, 71^. Increasingly, pharmaceutical companies are involved in multi-organization collaborations and public-private partnerships (PPP). To address these issues, Collaborative Drug Discovery, Inc. (CDD) created a software which enables researchers to have their own private vault for storing chemistry and biology data, which can be securely shared and mined while maintaining IP status ^72^. CDD itself has found a niche in hosting large-scale collaborations such as More Medicines for Tuberculosis (MM4TB), Bill and Melinda Gates Foundation (BMGF) TB Accelerator ^73^, and the NIH Blueprint for Neuroscience Research (BPN). In addition, rare disease research organizations, such as the Myelin Repair Foundation (MPF) and Jonah’s Just Begun, have used CDD to manage ongoing drug discovery efforts. CDD has a trove of public information, which provides datasets that can be useful for rare disease researchers. These include FDA approved drugs and compounds that have been identified by *in vitro* screening for repurposing ^74^, and the National Center for Advancing Translational Sciences (NCATS) molecules for repurposing ^75^. CDD has recently added the NIH's Molecular Libraries Probe Production Centers Network (MLPCN) probe compounds alongside the scoring of these molecules by an experienced medicinal chemist ^76^. Comparison of these public datasets with private data may lead to novel drug repositioning ideas, which may in turn mean an accelerated path towards new treatments ^74, 77, 78^. Many academic screening centers are focused on repurposing current FDA approved compounds ^79^, so the missing piece is developing phenotypic or target based screens for more rare diseases.

### Open Drug Discovery Teams (ODDT)

Beyond the desktop, we must seriously consider how mobile devices could be used to share data and foster collaborations in rare diseases. A mobile app called Open Drug Discovery Teams (ODDT) ^80, 81^ was created to collect Twitter feeds on multiple scientific hashtags (e.g., rare diseases like #huntingtons, and #sanfilipposyndrome, #gaucher, #huntersyndrome #fabry, #Tay-Sachs, #NGLY1, #hurlersyndrome, #pompedisease, #krabbe, #fmdaware, #niemannpick and #Batten). Collecting tweets and information from the web creates an open database for these diseases. The architecture of ODDT has been described recently ^82^, and the use and function of the app have also been discussed ^80^. The app is also small molecule aware so it can be used to share structures and activity data. The limited funding available for rare disease drug discovery and development suggests why we should be looking at alternative, lower cost approaches. ODDT could even become a useful assistant to scientists, small rare disease foundations, and advocates to help find collaborators or groups to fund. ODDT is ultimately a simple tool that uses Twitter for serious science applications that could be expanded in several directions to help the rare disease community. For example, besides collaboration towards one rare disease treatment, there is also opportunity for rare disease researchers to work together to compare HTS drug discovery data. Can rare disease groups learn from one another? Could hits found for related rare diseases have additional applications, or might safety and toxicity issues be determined earlier if the data were compared sooner?

## What is still needed

### Dissemination of information to patients, physicians and advocates that connects genetics to pharmacology

How can treatment options be identified and/or created in a patient relevant time frame? The most obvious way is to identify an approved medicine that could be repurposed, or if warranted, used off label. Another option is to discover a medicine specific for that disease. Currently only few medicines are discovered each year for rare diseases. A recent analysis showed only 46 first in class medicines approved for rare diseases in a 14 year period ^3^. With almost 7000 rare diseases, it is impossible to discover medicines for all of these using current research practices. Clearly, the number of identified human rare disease genes significantly outstrips the number of global research laboratories available to investigate a given disorder. The current productivity of drug discovery will never fill this need. Therefore, the development of a strategic toolbox and preclinical research pathway for inherited rare diseases has been proposed ^83^.

The unfortunate reality of drug discovery as it is currently practiced is it is a long and costly process. Increased success in rare disease drug discovery will require better diagnostics, an understanding of disease that provides good translational biomarkers, and clearer clinical development programs. The mechanisms underlying rare diseases are not well understood, patients are hard to identify and diagnose, and no regulatory precedent for the disease may exist, all of which makes designing and conducting drug development programs very difficult. There has been a dramatic increase in research and development spending without the corresponding increases in new medicines. The current trend is to spend more to increase knowledge, however this has not increased the clinical success rate. The low productivity is unacceptable for rare disease drug discovery. Funds need to be used more efficiently. The new knowledge needs to be used more effectively to identify treatment options. Solutions that provide for more treatment options in addition to new medicines are needed. Some hope in rare diseases is provided by understanding the genetic cause to the disease. As noted above, greater than 80% of rare diseases are due to a genetic defect. This understanding can focus research efforts and inform potential medical treatments. However, knowing the identity of a casual gene does not readily lead to a medicine that will cure the disease. Opportunities exist to use the genetic information to provide treatment options, in addition to informing drug discovery research.

 What are the options if there is not the time or funding available to discover and develop new medicines? One potential option is to identify molecules approved for human use including pharmaceuticals, nutraceuticals and herbal products that can be safely given to patients. While this seems obvious, there is a huge barrier to the dissemination of information between researchers and patients, physicians and advocates to develop a treatment plan based on all the available information. For rare disease researchers, a comprehensive data management system that consolidated the underlying genetic and protein causes of these disparate rare diseases would be hugely useful. Bringing these data together in a comprehensive database with information that reaches beyond just the underlying gene to other biological relevant information such as pathway analysis will be critical to researchers performing drug discovery on these disparate diseases. In parallel, the patient of a child with a rare or ultra-rare disease has few options in the USA as there is currently no single entity that covers all rare disease research and clinical translational work. Ideally, a comprehensive database would be presented to patients and advocates in a factual manner that is easy to understand, difficult to misinterpret, and could lead to connections with scientific and medical experts.

The assimilation and dissemination of knowledge from the many scientific areas important to medicine, including genetics, biology, chemistry and pharmacology, is challenging even for experts. Tools that provide this information to patients, physicians and advocates will be of value to help provide insights into new treatment options and to identify new opportunities. For example, providing information on approved medicines or remedies in which the pharmacology could be related to a specific physiological system and/or gene may identify new treatment options and/or new research directions. One approach to address this need is with an *in silico* database, in which the knowledge is easily used by both professionals and non-professionals. We envision that the identification of a new gene and the corresponding biology may provide insights into pharmacology that may be addressed with approved medicines. This knowledge could be of use to identify compounds for testing in animal and cellular studies. There is also the possibility of off label use in the patients with the proper monitoring, if no other treatment options are available.

It can be a challenge to match pharmacology with biology and genetics, especially for non-experts. Even domain experts in genetics and biology, may not know the corresponding pharmacology and vice versa. A database or collaborative network that specifically provides this information and access to experts will be of value to patients with rare diseases. For example, commercially available databases already exist that relate a gene to biological pathways with known pharmacology ^84^ and could help identify treatment options amongst FDA-approved drugs, nutraceuticals or other compounds.

The details of hereditary angioedema (HAE) provide a nice example of how the identification of the mutated genes led investigators to identify the biological systems involved, which in turn provided clues to potential pharmacological intervention and approved therapeutics ^85^. HAE is a rare genetic disorder that leads to episodes of extreme swelling caused by mutations to C1-esterase-inhibitor (C1-INH), a protease inhibitor that functions in the complement cascade in the immune system ^86^. HAE is characterized by low levels of C1-INH activity and low levels of C4 in the classical complement pathway ^87^. C1-INH regulates the activation of complement and intrinsic coagulation (contact system pathway), and is a major endogenous inhibitor of plasma kallikrein. The kallikrein-kinin system is a complex proteolytic cascade involved in the initiation of both the inflammatory and coagulation pathways. One critical aspect of this is the conversion of High Molecular Weight (HMW) kininogen to bradykinin by the protease plasma kallikrein. In HAE, normal regulation of plasma kallikrein activity and the classical complement cascade is not present. During attacks, unregulated activity of plasma kallikrein results in excessive bradykinin generation. Bradykinin is a vasodilator which is thought to be responsible for the characteristic HAE symptoms of localized swelling, inflammation, and pain ^88^. Two treatments for acute episodic attacks of HAE were developed once the causative gene was uncovered and required an in-depth understanding of the biology. Ecallantide binds to plasma kallikrein inhibiting the conversion of HMW kininogen to bradykinin ^89^. Icatibant is a competitive antagonist selective for the bradykinin B2 receptor ^90– 92^. Thus, this provides a clear example of how genetics can be connected to known pharmacology.

St. Jude Children’s Research Hospital, which from humble beginnings has transformed many cancers of children into treatable diseases through combined basic and translational research while at the same time becoming a world class center of excellence, offers a glimmering example or model for the rare disease community. Perhaps a dedicated rare disease institute to help facilitate and organize collaboration for translational research could be valuable. The development of such a center would need coordination between foundations, philanthropists, researchers, and government to ensure that it could become a reality.

It is our opinion that we need to centralize many of the rare disease efforts and translate findings to other rare diseases where there may not be current organizations driving the research. These efforts could include the development of databases of transcriptional profiles for thousands of compounds which many pharmaceutical companies have access to. Computational advances could be used in so many areas that would help rare disease research. This might include improving the prediction of small molecule-RNA/protein interactions, generalizing ADME-toxicology for oligonucleotides, or possibly identifying a druggable pathway that allows the persistence of higher levels of mutated and mis-folded protein. These may be just starting points for additional investments. With so many *in silico* cheminformatic and bioinformatic methods ^93, 94^, bringing them together via data integration platforms like those for systems biology, could help areas of research such as chaperonin identification ^74, 95^. Unfortunately, there is currently no definitive database for collaboration and education that disseminates available knowledge to rare disease stake holders (patients, physicians, advocates) in a usable/interpretable form. Such a database may provide insights into additional treatment options in a time frame relevant to patients.

An institute for rare diseases, which could be informatics driven, to centralize and direct the various ongoing academic collaborations funded by the rare disease groups would be a huge advance for optimal collaboration. Such an effort could use the various existing databases to mine for compounds as potential treatments for rare diseases. A recent effort to collate 456 FDA compounds approved for use in the pediatric population may be a starting point for repurposing these compounds for rare diseases computationally ^96^. An institute would partner with a center with HTS screening resources and would leverage existing infrastructure and researchers across many other institutes. Rare disease patient groups would be targeted to provide foundational funding and access to their complete researcher and patient networks. Pharmaceutical companies would be involved to provide access to compounds and databases for mining. In addition, advice from a scientific advisory board of experienced drug developers would be critical. The institute would share IP with the groups involved. The goal would be the creation of a world class center for rare diseases, becoming a magnet for global rare disease researchers, clinicians, patients, and companies, and it would be self-funding. The ultimate measure of its success would be how many treatments for rare diseases would be approved.

## A hopeful future

**Figure 1.**
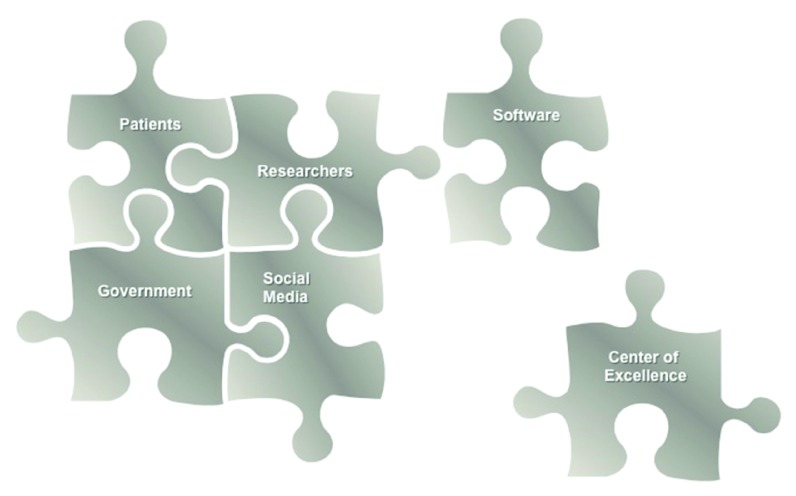
An illustration of the pieces of the rare disease jigsaw that could be brought together for developing treatments more efficiently.

The features of rare diseases that lead to their unique challenges can also become advantages for finding new therapeutics. Once united, a well-defined patient population, a defined genetic etiology, and a dedicated advocacy foundation can catalyze drug discovery. Collaboration between all of the key entities ( Figure 1), including academic institutions, government, biopharmaceutical companies, advocacy organizations, and non-profits is critical for moving rare disease drug discovery efforts forward. In addition, computational approaches can help foster the collaborations, add efficiency, build on previous efforts, and ultimately drive research in new directions. Individual rare disease researchers may also benefit from working together, perhaps through a centralized institute, to share resources towards the ambitious goal of finding treatments for the large unmet need.
