# Supplementary material for: Collaboration for rare disease drug discovery research
Source: F1000Res. 2014 Oct 31;3:261. [Version 1] doi: 10.12688/f1000research.5564.1 (PMC4314660; doi:10.12688/f1000research.5564.1)
Supplement: Peer review history

# Peer review: Referee response for version 1

**DOI:** 10.5256/f1000research.5946.r7513

**Published:** 2015-01-28

**Authors:** Stephen Groft

**Affiliations:** 1 Office of Rare Diseases Research, National Institutes of Health, Bethesda, MD, USA

This is a very well referenced opinion article on rare diseases and orphan products including historical and current activities that have led to an increased global emphasis on rare diseases research and orphan products development.  The article highlights several of the extremely important aspect of research and development for products for rare diseases – collaboration and coordination of activities.  Often, the coordination is led by the leadership of the patient advocacy groups.   Since very few research locations have sufficient patient populations to initiate patient recruitment sufficient to open and complete the study, a critical mass of investigators must be established from USA and international research sites. Likewise, patients and patient advocacy groups are now recognized key partners in the research continuum facilitating the recruitment of patients and providing active liaison services with the academic research community, biopharmaceutical industry, media, Government research and regulatory agencies, and the public.

The article highlights several rare diseases requiring coordinated activities leading to research advances for several rare diseases, e.g., Spinal Muscular Atrophy, Charcot-Marie-Tooth disease, Giant Axonal Neuropathy, Sanfilippo syndrome.  These disorders have unique requirements in research design and initiation of clinical trials with appropriate plans for data collection and analyses. The requirements necessitate early and constant contact with the regulatory agencies to reach agreement on biomarkers and clinical or surrogate endpoints for the study, the number of patients required for the study or clinical trial, and the analyses of data to be used to establish the safety and efficacy of the product prior to patient and physician access for patient care. This emphasis on expanded interactions with regulatory agencies has been presented in the article and is particularly important for consideration by the reader as FDA has added many unique regulatory approaches to product review, such as, priority and expedited review and approval of products, fast track review, and breakthrough therapy designation.  It is important to understand the value of these regulatory programs and benefits accompanying these products in the research and review stages of product development.

The authors’ opinion article indicates one of the great barriers to the development of orphan products has been the financial risk of developing a product for a disease with very small patient populations of potential users.  The collaborative method of research and development will spread the financial risk over many partners especially in the pre-clinical and early clinical stages of development.  For some rare diseases, industry partnerships have been easier to obtain after certain milestones have been exceeded and the likelihood of clinical success is apparent with limited or relative toxicity expected.  Reaching these milestones have been reported as significant in the decision making process.  For most rare diseases, sharing of global resources remains a powerful incentive for biopharmaceutical industry participation.

The authors conclude with suggested future activities including the dissemination of information to patients and clinicians about novel treatments of rare diseases with selected sub-population of patients with specific genetic mutations.  Also, the authors suggest a continued and expanded centralization of many of the rare disease activities including the development of an informatics driven initiative.  The authors provide a hopeful view of the future based on even greater global coordination and collaboration offered by centralized sources of information and readily available shared resources from multiple resources in the public and private sectors.

I have read this submission. I believe that I have an appropriate level of expertise to confirm that it is of an acceptable scientific standard.

# Peer review: Referee response for version 1

**DOI:** 10.5256/f1000research.5946.r6611

**Published:** 2014-11-18

**Authors:** Nicholas Meanwell

**Affiliations:** 1 Department of Chemistry, Bristol Myers Squibb Research and Development, Wallingford, CT, USA

This is a well-written and articulate article advocating collaborative research and organizational approaches to develop drugs with the potential to treat rare diseases. The case for collaboration is thoughtfully developed and the authors make compelling arguments.  I fully sympathize with the contentions laid out in the article and this piece will be a valuable addition to the literature.

I have read this submission. I believe that I have an appropriate level of expertise to confirm that it is of an acceptable scientific standard.
